# Inhibiting *CBP* Decreases AR Expression and Inhibits Proliferation in Benign Prostate Epithelial Cells

**DOI:** 10.3390/biomedicines11113028

**Published:** 2023-11-11

**Authors:** Xingxing Tang, Zhifu Liu, Zheng Li, Chenchen Huang, Wei Yu, Yu Fan, Shuai Hu, Jie Jin

**Affiliations:** 1 Department of Urology, Peking University First Hospital, Beijing 100034, China; tangxingxing@bjmu.edu.cn (X.T.);; 2 Institute of Urology, Peking University, Beijing 100034, China; 3 Beijing Key Laboratory of Urogenital Diseases (Male), Molecular Diagnosis and Treatment Center, National Research Center for Genitourinary Oncology, Beijing 100034, China

**Keywords:** *CBP*, androgen receptor, benign prostatic hyperplasia

## Abstract

(1) Background: CREB-binding protein (*CBP*) is a key transcriptional coactivator of androgen receptors (AR). We conducted this study to investigate the effects of *CBP* on AR expression and proliferation in benign prostatic hyperplasia (BPH) prostate epithelial cells. (2) Methods: By analyzing a published data set, we found that *CBP* was closely related to the gene expression of AR in prostate cells. We enrolled 20 BPH patients who underwent transurethral resection of the prostate (TURP) in Peking University First Hospital in 2022, and analyzed the expressions of *CBP* and AR in BPH prostate tissues. Then, we used ICG-001 and shRNA to inhibit *CBP* in prostate epithelial cells (BPH-1 cells and RWPE-1 cells), and conducted immunofluorescence, cell viability assay, flow cytometry analysis, and Western blot to analyze the effects of *CBP* on AR expression and proliferation in prostate epithelial cells. We also studied the interaction between *CBP* and AR through a co-immunoprecipitation assay. (3) Results: *CBP* is consistent with AR in expression intensity in prostate tissues. Inhibiting *CBP* decreases AR expression, and induces proliferation inhibition, apoptosis, and cell cycle arrest in BPH prostate epithelial cells. The co-immunoprecipitation assay showed that *CBP* binds with AR to form transcription complexes in prostate epithelial cells. (4) Conclusions: Inhibiting *CBP* decreases AR expression and inhibits proliferation in benign prostate epithelial cells. *CBP* may be a potential target to affect AR expression and the proliferation of prostate epithelial cells in BPH.

## 1. Introduction

Benign prostatic hyperplasia (BPH) is a common disease in elderly men [1]. Since pathogenic factors induce BPH by disrupting the meticulous balance between the proliferation and apoptosis of prostate epithelial cells [2], studying the regulation of proliferation and apoptosis in prostate epithelial cells is of great significance for BPH. Thus far, although various studies have reported that the GRP78, NELL2, METTL3/YTHDF2/PTEN signaling pathways, DNA methylation, and others may play roles in regulating the proliferation and apoptosis of prostate epithelial cells [3,4,5], androgens and androgen receptors (AR) remain the key in the proliferation and apoptosis of prostate epithelial cells in BPH [6]. AR signaling plays a key role in the occurrence and development of BPH [7], and blocking this signaling could reduce the prostate volume and relieve lower urinary tract symptoms (LUTS) caused by BPH [8]. Bauman and colleagues found that compared with normal prostate epithelial cells, the AR activity of BPH prostate epithelial cells is significantly increased [9]. Alonso-Magdalena et al. reported that AR could promote the progression of BPH through both directly promoting the proliferation of prostate epithelial cells and increasing epithelial–mesenchymal transition (EMT) [10]. Izumi et al. found that AR in prostate epithelial cells could promote the development of BPH through both epithelial-to-stromal crosstalk and EMT [7]; moreover, Lu and colleagues found that AR could also affect the development of BPH by inducing the inflammatory environment with macrophage infiltration [11]. Therefore, the expression of AR in benign prostate epithelial cells is closely related to the occurrence and development of BPH, and regulating AR expression in benign prostate epithelial cells may have positive significance in the treatment of BPH. However, the means by which AR expression and the proliferation of benign prostate epithelial cells are not fully elucidated.

Current studies show that the role of AR in prostate epithelial cells depends on a series of transcriptional coactivators, including *CBP* (CREB-binding protein), *SRC-1*, *ARA-70*, *TIP60*, etc. [12]. We used a publicly published data set [13] to study the effect of androgens on gene expression in prostate tissue, and found that *CBP*, a transcriptional coactivator of AR, is closely related to the gene expression of AR in prostate cells via bioinformatics analysis. Currently, several studies have shown that *CBP* is an important transcriptional coactivator of AR in prostate epithelial cells, and the realization of AR function depends on *CBP* [14]. The latest study has demonstrated that inhibiting *CBP* significantly decreases AR expression and effectively inhibits the proliferation of prostate cancer cells [15]. However, it is not fully understood whether *CBP* could also affect AR expression and the proliferation of prostate epithelial cells in BPH.

In this study, we found that inhibiting *CBP* effectively decreases the expression of AR, as well as induces proliferation inhibition, apoptosis, and cell cycle arrest in benign prostate epithelial cells. This suggests that *CBP* may be a potential target to affect AR expression and the proliferation of prostate epithelial cells in BPH.

## 2. Materials and Methods

### 2.1. Patients and Prostate Tissues

A total of 20 patients who underwent transurethral resection of the prostate (TURP) for BPH in Peking University First Hospital between July 2022 and October 2022 were enrolled. The patients were not treated with medication before surgery, and patients with prostatitis, urinary tract infections, or previous prostate surgery were excluded. The prostate specimens were examined microscopically by two pathologists to ascertain a diagnosis of BPH without prostatic intraepithelial neoplasia, prostate cancer, and prostatic stromal sarcoma. For each patient, we used the paraffin-embedded prostate specimen to make serial sections, and selected two closely adjacent sections for the immunohistochemical staining of *CBP* and AR, respectively, to observe whether *CBP* and AR were consistent in their expression intensity and localization in BPH prostate tissues. This study was conducted in accordance with the Declaration of Helsinki, and approved by the ethics committee of Peking University First Hospital.

### 2.2. Cell Culture

The human BPH prostate epithelial cell line BPH-1 and the human normal prostate epithelial cell line RWPE-1 were obtained from the American Type Culture Collection (ATCC, Manassas, VA, USA). The BPH-1 cells were cultured in DMEM medium containing 10% FBS and 1% penicillin/streptomycin, and the RWPE-1 cells were cultured in KM medium containing 1% KGS and 1% penicillin/streptomycin. All of the cells were cultured in a humidified incubator at 37 °C with 5% CO_2_. The cells in the control group were treated with 0.1% DMSO, and the cells in the experimental group were treated with the *CBP* inhibitor ICG-001 at doses of 15 μM and 30 μM, respectively. The processing time of the cell viability assays was 72 h, and the processing time of the other experiments was 48 h.

### 2.3. Construction of CBP Knockdown Cells

We constructed shRNA lentiviruses knocking down *CBP* (shCBP) and the negative control lentivirus (shNC). The BPH-1 cells were cultured in 6-well plates; 5 μL of lentivirus was added to each well, and fresh medium was replaced regularly. Cell passage was carried out when the cell abundance was about 90%, and a subsequent cell culture was carried out in a medium containing 2 mg/mL bleomycin. The knockdown effect of *CBP* was analyzed using Western blot; a reduction of over 70% in expression was considered effective.

### 2.4. Main Reagents and Instruments

The PBS was purchased from HyClone. The KM Medium was purchased from ScienCell. FBS (04-001-1ACS) was purchased from BI. DMEM (C11995500BT), penicillin/streptomycin antibiotic (15140-122), and 0.25% trypsin-EDTA (25200-056) were purchased from Life Technologies Corporation. Cyclin B1 (ab32053) and CDK1 (ab133327) antibodies were purchased from Abcam. Cleaved caspase-3 (9661), c-Myc (5605), and Bax (2772) antibodies were purchased from CST. β-tubulin antibody (HC101-01) was purchased from Transgen. Sheep anti-rabbit antibody (RS0002) and sheep anti-mouse antibody (RS0001) for the Western blotting studies were purchased from ImmunoWay. ICG-001 (S2662) were purchased from Selleck. The immunohistochemistry set (PV-6000) was purchased from Zhongshan Jinqiao. The cell cycle detection kit (KGA511) was purchased from Keygen Biotech. ECL (WBKLS0500) was purchased from EMD Millipore. The flow cytometer (LSR Fortessa X-20) was purchased from BD. The fluorescence microscope (IX71) was purchased from Olympus. The confocal microscope (TCS-SP8 DIVE) was purchased from Leica. The gel imaging system (GeneGnome XRQ) was purchased from Syngene.

### 2.5. Immunohistochemistry Staining and Quantitative Analysis

The sections were deparaffinized in xylene solution and rehydrated using graded concentrations of ethanol. After antigen retrieval, the activity of endogenous peroxidase was blocked for 30 min, then the primary antibody was added to incubate overnight at 4 °C. The secondary antibody was added the next day. After mounting, the slides were observed and photographed using a microscope.

In terms of the quantitative analysis for *CBP*, we selected three typical areas containing prostate epithelial cells for each slide and obtained the images. For each image, we used the color deconvolution (HDAB) function of ImageJ v1.53 (NIH, Bethesda, MD, USA) to obtain the HE staining (blue purple) and *CBP* staining (brown) images. For the *CBP* staining, it was mainly concentrated in the nuclei of the prostate epithelial cells; thus, we firstly selected and labeled 10 nuclei of prostate epithelial cells randomly to confirm the measurement areas on the HE staining images, then circled the same nuclei on the *CBP* staining images and measured the staining intensity of the *CBP*. The mean of these 10 staining intensity values was the mean intensity of the *CBP* for this image, and all of the images needed to be measured. For the AR, the same process was conducted. Finally, we used the mean intensity of the *CBP* and the mean intensity of the AR to quantitatively analyze their expression intensities and correlation in prostate epithelial cells.

### 2.6. Immunofluorescence

The cells were grown on glass slides, fixed with paraformaldehyde, and incubated with 0.5% Triton X-100. Then, the cells were blocked with 1% BSA and incubated overnight at 4 °C with primary antibody. The next day, the fluorescent secondary antibody was added, and the sections were mounted with mounting medium and DAPI. Finally, we observed and photographed the sections under a confocal microscope.

### 2.7. Cell Viability Analysis

The cell viability was determined using CCK8 assays. Plates with 96 wells were used, 1000 BPH-1 cells or 3000 RWPE-1 cells were implanted per well, and five replicate wells were set up. We added 100 μL of CCK8 to each well, incubated them at 37 °C for 2 h when measuring, and then used a microplate reader to detect the OD450 values.

### 2.8. Cell Apoptosis Analysis

Cell apoptosis was detected using annexin V and the propidium iodide (PI) double-staining method. The cells were digested with EDTA-free trypsin and washed with PBS, then fluorescent dye-labeled annexin V and PI were added. After treatment for at least 10 min, the cells were analyzed using flow cytometry.

The results included cells in four quadrants. The upper left (UL) quadrant Q1 represents dead cells (annexin V−/PI+); the upper right (UR) quadrant Q2 represents late apoptotic cells (annexin V+/PI+); the lower right (LR) quadrant Q3 represents early apoptotic cells (annexin V+/PI−); and the lower left (LL) quadrant Q4 represents normal (living) cells (annexin V−/PI−). In this study, we included early apoptotic cells (Q3) and late apoptotic cells (Q2) as apoptotic cells.

### 2.9. Cell Cycle Analysis

The cells were digested with trypsin and stored in 70% ethanol overnight. The next day, the cells were washed with PBS and PI dye was added; then, they were analyzed using flow cytometry.

### 2.10. Western Blot

After treating the cells with protein lysate, the supernatant was collected via centrifugation. Then, an appropriate amount of protein loading buffer (PLB) was added to prepare the protein sample solution. Each well was loaded with 10 μg of protein, and subjected to electrophoresis on a 10% SDS-PAGE gel. Subsequently, the proteins were transferred onto a PVDF membrane using electroporation. After blocking the PVDF membrane with milk, it was incubated overnight at 4 °C with a primary antibody diluted to a ratio of 1:1000. The next day, the membrane was incubated with a secondary antibody diluted to a ratio of 1:10,000 for 1 h, followed by the addition of an ECL substrate. Finally, the membrane was visualized using a gel imaging system.

### 2.11. Co-Immunoprecipitation (CoIP)

After centrifugation of the lysed cells, the supernatant was collected and then incubated overnight at 4 °C with primary antibody or IgG antibody, which was incubated with magnetic beads at room temperature for 1 h the next day. Subsequently, PLB was added, and the mixture was heated at 100 °C for 10 min. At last, the protein sample solution was obtained by separating the magnetic beads using a magnetic rack. The subsequent steps used were the same as for the Western blot analysis.

### 2.12. Statistics

The bioinformatics analysis and the generation of related figures were performed using R language v4.1.0. The protein expression intensity in IHC and cell fluorescence intensity in immunofluorescence were measured using ImageJ v1.53 (NIH, Bethesda, MD, USA). The cell cycle data were analyzed using Modfit LT v5.0 (Verity Software House, Topsham, ME, USA). The cell apoptosis data were analyzed using FlowJo v10.4 (Ashland, OR: Becton, Dickinson and Company, Franklin Lakes, NJ, USA). Other statistical analyses and the generation of related figures were performed using GraphPad Prism v9.3.1 (GraphPad Software, La Jolla, CA, USA). All of the data are presented as mean ± standard deviation. One-way analysis of variance (ANOVA) was used for data comparison among multiple groups, and Pearson’s test was used for correlation analysis. A *p*-value < 0.05 was considered statistically significant.

## 3. Results

### 3.1. The Gene Expressions of CBP and AR Were Closely Related

**Figure 1:**
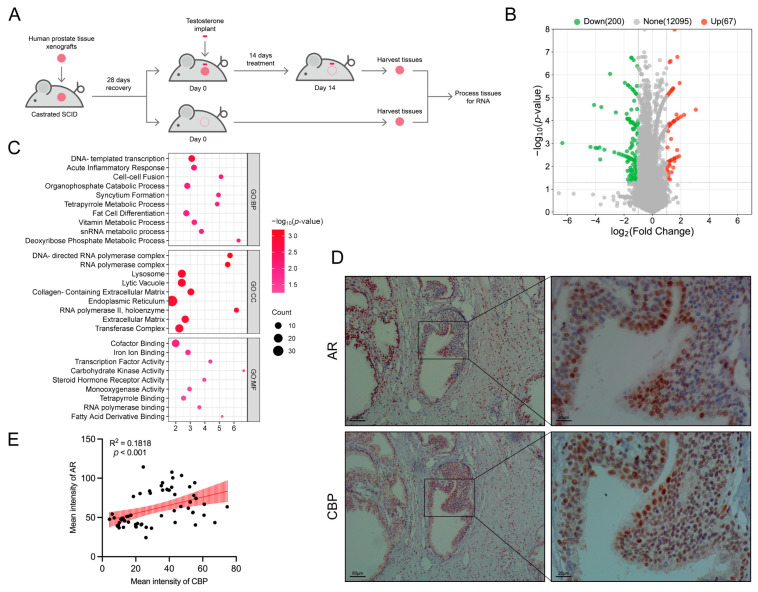
*CBP* and AR gene expression are closely related in BPH prostate cells. (**A**) Study design of Love and colleagues (part). SCID mice and human BPH tissues were used to construct PDX animal models; testosterone rods were implanted into mice, and blank control group was set up to observe the effect of androgen on gene expression in BPH prostate cells. (**B**) Volcano plot of gene expression differences between androgen-treated prostate cells and non-androgen-treated prostate cells. (**C**) Enrichment analysis results. (**D**) Immunohistochemical staining of AR and *CBP* in BPH prostate tissues. (**E**) Scatter diagram of correlation of expression intensity, and the red area represents the 95% confidence interval of the fitted line. SCID, severe combined immunodeficient; PDX, patient-derived xenograft; GO, gene ontology; BP, biological process; CC, cellular component; MF, molecular function.

Using the GEO database, we retrieved a study in which Love and colleagues constructed BPH animal models using severe combined immunodeficient (SCID) mice and human BPH tissues. Testosterone implants were implanted into the mice, and a blank control was set up to observe the effects of androgen on gene expression in BPH prostate cells (Figure 1A). We performed a bioinformatics analysis on their data, and the results showed that compared to prostate cells not treated with androgens, 200 genes were significantly downregulated (*p* < 0.05), and 67 genes were significantly upregulated (*p* < 0.05) in the androgen-treated prostate cells (Figure 1B). The enrichment analysis showed that these differentially expressed genes were enriched in DNA transcription-related pathways (Figure 1C). Further protein interaction analysis identified hub genes that are closely associated with AR gene, with *CBP* ranking third (Table 1).

### 3.2. CBP and AR Were Consistent in Expression Intensity in BPH Prostate Tissues

IHC staining of BPH prostate tissue showed abundant expressions of *CBP* and AR in the prostate tissues, and both *CBP* and AR were mainly expressed in prostate epithelial cells. At the same time, it could be observed that the expressions of *CBP* and AR were consistent in intensity in the prostate tissues. The quantitative analysis showed a significant positive correlation between the expression intensity of *CBP* and AR in benign prostate epithelial cells (R^2^ = 0.1818, *p* < 0.001) (Figure 1D,E).

### 3.3. Construction of CBP Knockdown Cells

**Figure 2:**
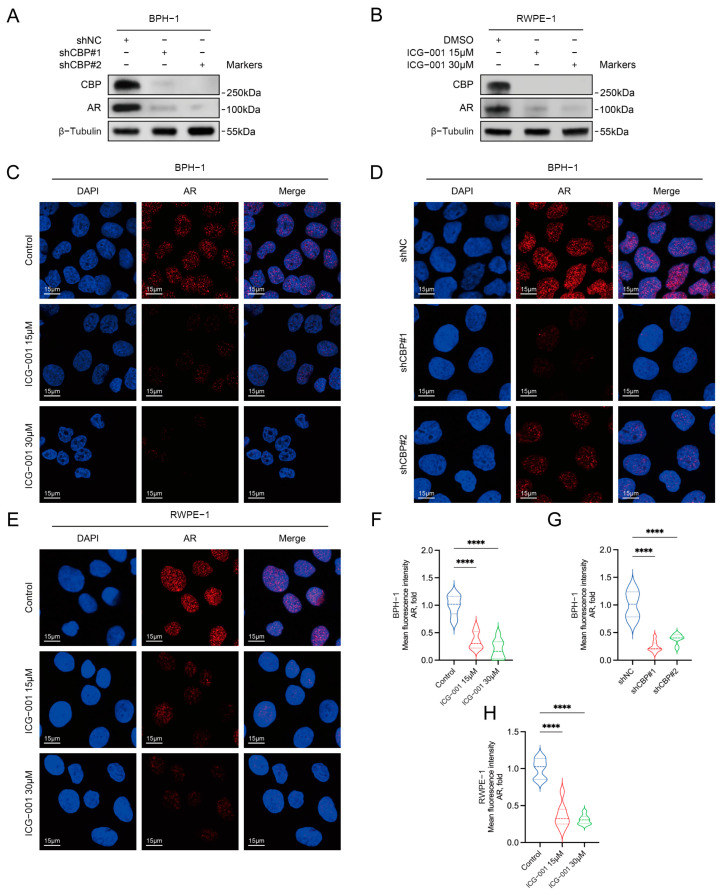
Inhibiting *CBP* decreases AR expression in benign prostate epithelial cells. (**A**) Western blot was used to detect the change in AR level after shRNA was used to knock down *CBP* in BPH-1 cells. (**B**) Western blot was used to detect the change in AR level after ICG-001 was used to inhibit *CBP* in RWPE-1 cells. (**C**) Immunofluorescence staining was used to detect changes in AR levels after using ICG-001 or (**D**) shRNA to inhibit *CBP* in BPH-1 cells, and blue represents DAPI, red represents AR; (**F**,**G**) Quantitative analyses diagrams. (**E**) Immunofluorescence staining was used to detect the change in AR level after ICG-001 was used to inhibit *CBP* in RWPE-1 cells, and blue represents DAPI, red represents AR; (**H**) Quantitative analysis diagram. DMSO, dimethyl sulfoxide; DAPI, 4’,6-diamidino-2-phenylindole; **** *p* < 0.0001.

We constructed shRNA lentiviruses knocking down *CBP* (shCBP#1 and shCBP#2) and the negative control lentivirus (shNC); using these lentiviruses, we constructed *CBP* knockdown BPH-1 cells (shCBP#1 cells and shCBP#2 cells) and the negative control BPH-1 cells (shNC cells). Western blot results showed that compared with the shNC cells, the expressions of *CBP* in the shCBP#1 cells and shCBP#2 cells were significantly reduced (Figure 2A), suggesting that the *CBP* knockdown cells were successfully constructed.

### 3.4. Inhibiting CBP Decreased the Expression of AR in Prostate Epithelial Cells

The Western blotting results reveal that, compared with the control group, both BPH-1 cells and RWPE-1 cells showed a significant decrease in AR expression after using ICG-001/shCBP to inhibit/knockdown *CBP* (Figure 2A,B). The immunofluorescence results show that for BPH-1 cells, compared with the control group, the fluorescence intensity of AR was significantly reduced after using ICG-001/shCBP to inhibit/knockdown *CBP* (Figure 2C,D,F,G). For RWPE-1 cells, compared with the control group, the fluorescence intensity of AR was significantly decreased after using ICG-001 to inhibit *CBP* (Figure 2E,H).

### 3.5. Inhibiting CBP Induced Proliferation Inhibition in Prostate Epithelial Cells

**Figure 3:**
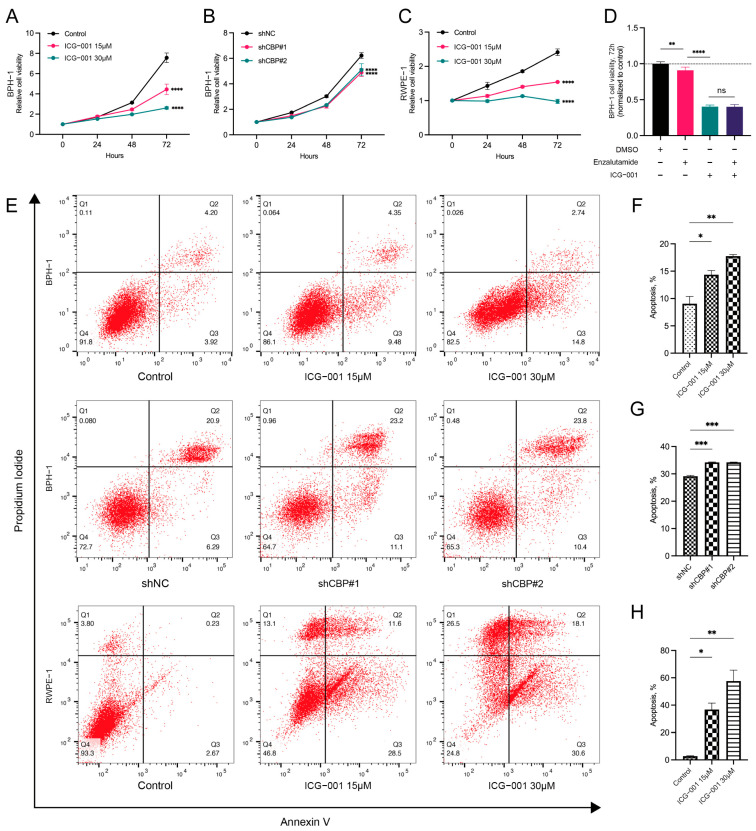
Inhibiting *CBP* inhibits proliferation and induces apoptosis in benign prostate epithelial cells. (**A**) CCK8 assay was used to detect the effect of different concentrations of *CBP* inhibitor (ICG-001) on the proliferation of BPH-1 cells and (**C**) RWPE-1 cells. (**B**) CCK8 assay was used to detect the effect of knocking down *CBP* with shRNA on the proliferation of BPH-1 cells. (**D**) Effects of AR antagonist (enzalutamide) and *CBP* inhibitor (ICG-001) alone/combined on the proliferation of BPH-1 cells. (**E**) Flow cytometry was used to detect the effects of inhibiting *CBP* (using ICG-001 or shRNA) on apoptosis of BPH-1 cells and RWPE-1 cells, as well as (**F**–**H**) the quantitative analyses diagrams. DMSO, dimethyl sulfoxide; ns, no significance; * *p* < 0.05, ** *p* < 0.01, *** *p* < 0.001, **** *p* < 0.0001.

The CCK8 assay measures changes in cell viability (cell number) that result from cell proliferation. We measured the changes in cell viability at four time points (0 h, 24 h, 48 h, 72 h), and found that the cell viability in the *CBP* inhibition group was significantly lower than that in the control group at 72 h, suggesting that inhibiting *CBP* could induce proliferation inhibition in BPH-1 cells and RWPE-1 cells, and the extent of inhibition is correlated with the concentration of ICG-001 (Figure 3A,C). Compared with the control group, knocking down *CBP* using shCBP induced proliferation inhibition of BPH-1 cells as well (Figure 3B).

Compared with the control group, using AR inhibitor enzalutamide or *CBP* inhibitor ICG-001 alone or in combination significantly induced the proliferation inhibition of BPH-1 cells (*p* < 0.01, *p* < 0.0001, *p* < 0.0001, respectively). There was no significant difference in the extent of proliferation inhibition of BPH-1 cells between using ICG-001 alone and using it in combination with enzalutamide (Figure 3D), indicating that inhibiting AR could not further inhibit the proliferation of prostate epithelial cells in the presence of *CBP* inhibition. These results suggest that the inhibitory effect of *CBP* encompasses the inhibitory effect of AR on prostate epithelial cells proliferation.

### 3.6. Inhibiting CBP Induced the Apoptosis of Prostate Epithelial Cells

Compared with the control group, inhibiting *CBP* with ICG-001 significantly increased the apoptosis of BPH-1 cells and RWPE-1 cells, and the extent of the apoptosis was correlated with the concentration of ICG-001 (*p* < 0.05, *p* < 0.01, *p* < 0.05, *p* < 0.01, respectively) (Figure 3E,F,H). Compared with the control group, knocking down *CBP* with shCBP significantly increased the apoptosis of BPH-1 cells as well (*p* < 0.05, *p* < 0.01, respectively) (Figure 3E,G).

### 3.7. Inhibiting CBP Induced Cell Cycle Arrest of Prostate Epithelial Cells

**Figure 4:**
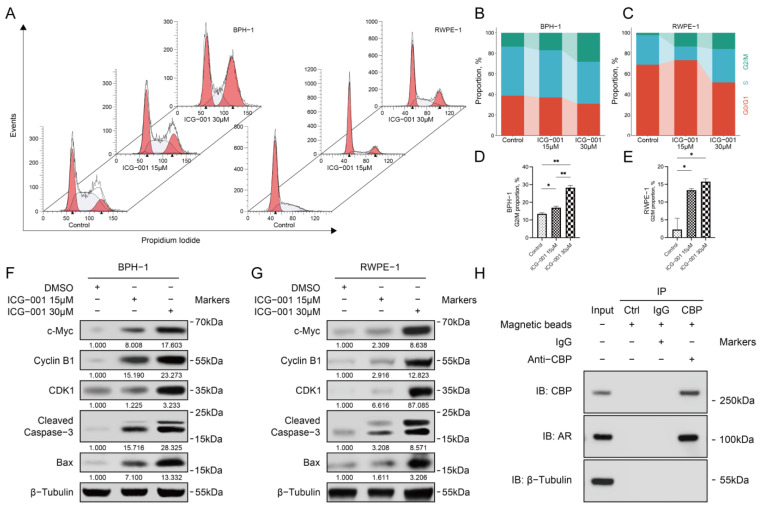
Inhibiting *CBP* induces cell cycle arrest and protein expression changes in benign prostate epithelial cells. (**A**) Using flow cytometry to analyze the effect of *CBP* inhibitor (ICG-001) on the cell cycle of BPH-1 cells and RWPE-1 cells; (**B**–**E**) Quantitative analyses diagrams. (**F**) Western blots were used to detect the effects of different concentrations of *CBP* inhibitor (ICG-001) on the expressions of apoptosis-related proteins and cell cycle-related proteins in BPH-1 cells and (**G**) RWPE-1 cells. (**H**) CoIP assay was used to detect the binding of *CBP* and AR in BPH-1 cells. DMSO, dimethyl sulfoxide; IP, immunoprecipitation; IB, immunoblot; Ctrl, control; * *p* < 0.05, ** *p* < 0.01.

Compared with the control group, inhibiting *CBP* with ICG-001 significantly increased the proportion of G2/M arrest cells in BPH-1 cells and RWPE-1 cells, and the extent of cell cycle arrest was correlated with the concentration of ICG-001 (*p* < 0.05, *p* < 0.01, *p* < 0.05, *p* < 0.05, respectively) (Figure 4A–E).

### 3.8. Inhibiting *CBP* Increased the Expressions of Apoptosis-Related Proteins and Cell Cycle-Related Proteins in Prostate Epithelial Cells

Compared with the control group, inhibiting *CBP* with ICG-001 increased the expressions of apoptosis-related proteins, including cleaved caspase-3, Bax, c-Myc, and cell cycle-related proteins including CDK1 and cyclin B1 in BPH-1 cells and RWPE-1 cells. The extent of the increases in cleaved caspase-3, Bax and c-Myc were correlated with the concentration of ICG-001 (Figure 4F,G).

### 3.9. CBP Combined with AR to Form the Transcription Complex in Prostate Epithelial Cells

The BPH-1 cell protein was extracted for the CoIP experiment, and the input protein included *CBP*, AR, and β-tubulin. AR in the precipitate were detected after using antibodies to bind *CBP* and using magnetic beads to precipitate them, suggesting *CBP* combines with AR to form the transcription complex in BPH-1 cells. While using magnetic beads or IgG antibody alone, neither *CBP* nor AR could be detected in the precipitate, excluding the non-specific binding of magnetic beads/IgG antibody and *CBP*/AR (Figure 4H).

## 4. Discussion

**Figure 5:**
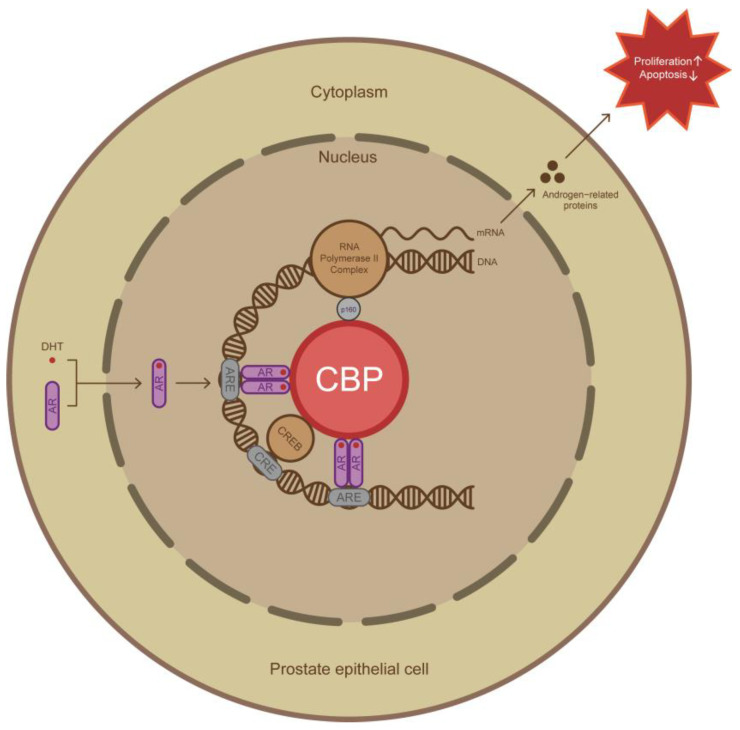
Schematic diagram of the transcription complex formed by *CBP* and AR in benign prostate epithelial cells. After AR activation, a dimer is formed and enters the nucleus, located in the ARE region of the target gene; it forms a transcription complex with transcriptional coactivators including *CBP*, and transcribes androgen-related proteins that promote proliferation and decrease apoptosis in benign prostate epithelial cells. The drawing of this schematic refers to other studies, and only transcriptional coactivators mentioned in this study are indicated. DHT, dihydrotestosterone; ARE, androgen response element; CRE, CREB response element.

Androgens play a key role in BPH [16], and the function of AR depends on various transcriptional coactivators such as *CBP*, *BRCA1*, *SRC1*, and so on [12], which form the transcription complexes with AR to ensure its function. The absence of transcriptional coactivators could lead to the inability of AR to form transcription complexes, leading to a loss in AR function [17], and free AR subsequently becoming completely degraded within 4–8 h [18]. Therefore, it is theoretically feasible to decrease the expression of AR by inhibiting transcriptional coactivators. Through bioinformatics analysis of the data set publicly published by Love and colleagues [13], we found that the transcriptional coactivator *CBP* of AR was closely related to AR in gene expression. *CBP* is a transcriptional coactivator that plays an important role in many crucial signaling pathways [19]. *CBP* possesses histone acetyltransferase activity [20], and by acetylating the lysine at the end of the histones, it changes the nature of the charge of histones, leading to a weakened interaction between DNA and histones, which in turn contributes to the occurrence of DNA transcription [14]. In addition, *CBP* is also closely related to histone methylation, and the *CBP*-associated histone methyltransferases (HMT) are specific for the lysines of histones. Histone methylation is a post-translational modification (PTM) that can either activate or repress gene expression [21]. Therefore, *CBP* may play an important role in the transcriptional regulation of AR-related genes by methylating histones. Multiple studies have reported that *CBP* plays a key role in assembling AR transcription complexes [22,23,24]. AR activated by DHT enter the nucleus, form a dimer that forms the transcription complex with other transcriptional coactivators such as *CBP*, CREB, and P160, and then transcribes androgen-related proteins that induce proliferation and decrease apoptosis in benign prostate epithelial cells later (Figure 5) [22,23,24]. The absence of *CBP* could lead to the inability of AR to form transcriptional complexes, leading to AR dysfunction. Recently, Welti et al. found that inhibiting *CBP* could induce AR expression downregulation and proliferation inhibition effectively in prostate cancer cells [15], and we also found *CBP* and AR were consistent in their expression intensities and localizations in BPH prostate tissues, indicating a potential correlation between AR and *CBP* in benign prostate epithelial cells.

Next, we analyzed the effect of *CBP* on the expression of AR in benign prostate epithelial cells. The results show that inhibiting *CBP* significantly decreases the expression of AR in prostate epithelial cells, and the extent of the decrease is positively correlated with the concentration of the *CBP* inhibitor ICG-001, suggesting that the maintenance of AR expression in prostate epithelial cells depends on *CBP*. Through the CoIP assay, it was intuitively observed that AR and *CBP* formed the transcriptional complex in prostate epithelial cells, which was consistent with the results of previous research; that is, the maintenance of AR expression and function depends on the presence of *CBP* [12].

Subsequently, we studied the effect of *CBP* on the proliferation of prostate epithelial cells, and the results show that inhibiting *CBP* could induce proliferation inhibition and apoptosis in prostate epithelial cells, and increase the expression of apoptosis-related proteins Bax, cleaved caspase-3, and c-Myc. Thus, in this study, we not only observed that inhibiting *CBP* significantly decreases AR expression in prostate epithelial cells, but also found that inhibiting *CBP* significantly inhibits proliferation in prostate epithelial cells. Considering that the prostate is an androgen-dependent organ, and androgens play an important role in the proliferation of prostate epithelial cells [16], we speculate that the inhibitory effect of inhibiting *CBP* on prostate epithelial cell proliferation may be partly achieved by inhibiting the expression of AR. We also observed in the experiment that the inhibitory effect of inhibiting *CBP* on prostate epithelial cells proliferation was much higher than inhibiting AR alone, which suggests that inhibiting *CBP* affects the proliferation of prostate epithelial cells through some other pathways, not just the AR-related signaling pathway. Eguchi et al. reported that this may be related to the WNT/β–catenin pathway [25]. Activation of WNT signaling inactivates GSK-3β and prevents the phosphorylation of β-catenin, which then accumulates in the cytoplasm and enters the nucleus to transcribe *CBP* to form the transcription complex that mediates a series of gene expressions that promote proliferation and differentiation [26]. Inhibition of *CBP* may reduce β-catenin transcriptional activity, thereby affecting the function of the WNT signaling pathway and inhibiting cell proliferation [25]; however, we did not analyze the effect of inhibiting *CBP* on the WNT/β–catenin signaling pathway in this study, so further in-depth exploration may be necessary. Overall, more experiments are needed to further clarify whether inhibiting *CBP* inhibits the proliferation of prostate epithelial cells by decreasing AR expression, and it may be more convincing to conduct rescue experiments by overexpressing *CBP* and knocking down AR to study the relationship between *CBP*, the AR signaling pathway, and cell proliferation in benign prostate epithelial cells. Considering that CBP has a high expression intensity in most cells, and has a molecular weight of about 300 kDa [27], there may be some technical difficulties in overexpressing *CBP* in prostate epithelial cells. Nevertheless, further exploration of the relationship between inhibiting the *CBP*-induced decrease in AR expression and proliferation inhibition in prostate epithelial cells is needed in the future.

We also analyzed the effects of *CBP* on the cell cycle of prostate epithelial cells. The results reveal that inhibiting *CBP* induces cell cycle arrest in the G2/M phase, and increases the expressions of G2/M phase-related cyclins CDK1 and cyclin B1. In the normal cell cycle, cyclin B1 binds and activates CDK1, and then CDK1 promotes cells to enter mitosis by phosphorylating related proteins (such as RPS3) [28]. The expression levels of CDK1 and cyclin B1 increase in the G2 phase and gradually degrade after mitosis, and CDK1 and cyclin B1 accumulate in the cell because they cannot be degraded when cells are arrested in the G2/M phase [28]. Therefore, we thought that the increased expressions of CDK1 and cyclin B1 may be a result of inhibiting *CBP*-mediated cell cycle arrest, while the mechanism needs to be further clarified.

In addition, it should be noted that *CBP* has a homologous protein P300, and the similarity between the two is as high as 63% [29]. P300 also has histone acetyltransferase activity, and parts of its functions are the same as those for *CBP* [30]. However, we did not analyze the effect of P300 on AR expression and the proliferation of prostate epithelial cells in this study. The ICG-001 we used in this study highly selectively inhibits *CBP*, and has nearly no effect on P300 [25]. Nevertheless, P300 is not without research value. Fu and his colleagues found that P300 is also a coactivator of AR, and P300 mutations can decrease ligand-dependent AR activation [31]. Therefore, the inhibition of P300 and the inhibition of *CBP* may have similar effects in prostate epithelial cells. However, since the protein interaction analysis results showed that *CBP* rather than P300 has a high connectivity with AR among genes affected by AR, we mainly focused on *CBP* in this study. Investigating the effect of P300 on AR expression and the proliferation of prostate epithelial cells, and the difference between it and *CBP* may be the next research direction.

Furthermore, certain limitations must be acknowledged in this study. Firstly, the sample size of enrolled patients is small (*n* = 20), which may not fully represent the expressions of *CBP* and AR in BPH prostate tissues. Secondly, the lack of animal experiments prevents us from further understanding the impacts of inhibiting *CBP* on AR expression and the proliferation of benign prostate epithelial cells in vivo, and on the progression of BPH in animals as well. Thirdly, in the histological study, there was a lack of non-BPH prostate tissues as a control, so it is impossible to observe whether the expressions of AR and *CBP* in BPH prostate tissues change compared with those in normal prostate tissues. Fourthly, as mentioned above, WNT, GSK-3β, and β-catenin may also be involved in the regulation of prostate epithelial cell proliferation by *CBP*, but they were not explored in this study. Fifthly, as mentioned previously, the *CBP* homolog protein P300 was not studied in this study. However, these limitations serve as areas for improvement in our future research.

## 5. Conclusions

In summary, this study indicates that inhibiting *CBP* decreases AR expression and induces proliferation inhibition, apoptosis, and cell cycle arrest in benign prostate epithelial cells. *CBP* may be a potential target to affect AR expression and the proliferation of prostate epithelial cells in BPH.

## Figures and Tables

**Table 1:** Top 10 hub genes with higher degrees of connectivity.

Rank	Gene Symbol	Gene Description	Degree
1	*GAPDH*	Glyceraldehyde-3-phosphate dehydrogenase	29
2	*TNF*	Tumor necrosis factor	25
3	*CBP*	CREB-binding protein	17
4	*PPARG*	Peroxisome proliferator-activated receptor, gamma	14
5	*POLR2C*	Polymerase (RNA) II polypeptide C	12
6	*TBP*	TATA box-binding protein	12
7	*STAT5A*	Signal transducer and activator of transcription 5A	10
8	*SERPINA1*	Serine proteinase inhibitor, clade A	10
9	*SAA1*	Serum amyloid A1	10
10	*CXCL10*	Chemokine (C-X-C motif) ligand 10	10

## Data Availability

All of the data sets generated in this study are available on reasonable request to the corresponding author.
